# A low proportion of HBeAg among HBsAg-positive pregnant women with known HIV status could suggest low perinatal transmission of HBV in Cameroon

**DOI:** 10.1186/1743-422X-9-62

**Published:** 2012-03-08

**Authors:** Anfumbom KW Kfutwah, Mathurin Cyrille Tejiokem, Richard Njouom

**Affiliations:** 1 Virology Service, Centre Pasteur du Cameroun, Membre du Réseau International des Instituts Pasteur, BP 1274 Yaounde, Cameroon; 2 Epidemiology Service, Centre Pasteur du Cameroun, Membre du Réseau International des Instituts Pasteur, BP 1274 Yaounde, Cameroon

**Keywords:** Hepatitis B virus, Prevalence, HBsAg, HBeAg, HIV, Cameroon, Pregnancy

## Abstract

**Background:**

Transmission of hepatitis B virus (HBV) from HBV-positive mothers to their infants is common and usually occurs when the mother is hepatitis B e antigen (HBeAg) positive and/or has a high HBV DNA load. In this study, we determined the prevalence of hepatitis B surface antigen (HBsAg) and HBeAg among pregnant women with known HIV status.

**Findings:**

A total of 650 pregnant women with a mean age of 26.2 years including 301 HIV-positives and 349 HIV-negatives were screened for HBsAg (Monolisa AgHBs Plus Biorad, France). Among the HBsAg-positives, HBeAg and anti-HBe were tested (Monolisa Ag HBe Plus Biorad, France). Overall, 51 (7.85%) were positive for HBsAg. The prevalence of HBsAg was not statistically different between HIV-positive and HIV-negative pregnant women [28/301 (9.3%) vs 23/349 (6.59%); p = 0.2]. None of the 45 HBsAg-positive samples was reactive for HBeAg.

**Conclusions:**

Our study indicates a high prevalence of HBsAg with very low proportion of HBeAg in Cameroonian pregnant women. Since perinatal transmission of HBV is mostly effective when the mother is also HBeAg-positive, our data could suggest that perinatal transmissions play a minor role in HBV prevalence in Cameroon. In line with previous African studies, these findings further suggests that horizontal transmission could be the most common mechanism of HBV infections in Cameroon.

## Introduction

Perinatal transmission of Hepatitis B virus (HBV) from infected mothers to their children is suspected to represent a major mode of disease transmission in high-prevalence areas [1]. HBV infection can be passed from infected mothers to their infants during pregnancy, at the time of birth, or after birth with most transmissions occurring during birth or in the perinatal period [2]. The risk of vertical transmission and resulting chronic infection from a chronic carrier mother (i.e. hepatitis B surface antigen (HBsAg) positive) to her infant is approximately 90% in hepatitis B "e" antigen (HBeAg) positive pregnant women with high HBV DNA titres [3-6].

The introduction of HBV testing in all pregnant women combined with immunoglobulin prophylaxis and/or hepatitis B vaccination immediately after delivery in all children born to HBsAg positive mothers have been reported to be useful strategies to reduce the prevalence of hepatitis B virus infection [7]. These actions would have the greatest impact in reducing the number of new hepatitis B carriers since perinatal transmission plays an important role in the maintenance of the reservoir of chronic HBV infections in areas of high prevalence of HBsAg ( ≥ 8%) like Cameroon.

Due to the shared modes of transmission, co-infections with HBV and HIV are common. The importance of implementing appropriate prophylactic care and follow-up of pregnant women infected with HBV is fundamental. Furthermore it is equally important to identify the HIV/HBV co-infected pregnant women since infected pregnant women are put on HIV prophylaxis to prevent mother-to-child transmission (MTCT) of HIV without information on their HBV status.

The aim of this study therefore was to determine the prevalence of hepatitis B surface antigen (HBsAg) among pregnant women with known HIV status in Yaounde, Cameroon.

## Study design and laboratory analyses

This study included pregnant women who participated in a Public Health Pilot Program (PHPP) for the Prevention of mother-to-child transmission of HIV-1 [8] and HCV [9] in Yaounde, Cameroon. Patients and Methods have been described elsewhere [8,9]. Briefly, between January 2000 and April 2003 these women were attending antenatal care at the mother and child center of the "Fondation Chantal Biya" (FCB) in Yaounde. These studies were approved by the National Ethics Committee and local health authorities in Cameroon and they also received the consent of the participating pregnant women. The women were screened for HIV as reported elsewhere [8]. In this study therefore, we consecutively selected 650 samples consisting of 301 HIV-positive and 349 HIV-negative samples from pregnant women. The HIV positive samples were selected within the period running from January 2000 to April 2003 while the HIV negative samples were selected from January to July 2000. The two groups were tested for HBsAg (Monolisa AgHBs Plus Biorad, France). This is a one step enzyme immunoassay technique of the 'sandwich' type. HBsAg positive samples were further tested for the presence of HBeAg and hepatitis B 'e' antibodies (anti HBe) respectively (Monolisa HBe Plus Biorad, France). The detection of HBeAg is based on a 2-step 'sandwich' enzyme immunoassay. For the detection of anti HBe, the same solid phase as for HBeAg was used. The test is based on competition between the immobilised antibodies present in the sample, towards a limited quantity of HBeAg used as a neutralising reagent (Monolisa HBe Plus Biorad, France).

## Results

The mean age (n = 550) in this study was 26.17 years (95% CI 25.7-26.6) with comparable values between the two groups: mean of 26.66 years (95% CI: 26.02-27.29 years) for HIV infected (n = 250) versus mean of 25.76 years (95% CI:25.12-26.40 years) for HIV uninfected (n = 300) women. A total of 51 (7.85%) out of the 650 pregnant women were found positive for HBsAg. Among these, 28/301 (9.3%) HIV-positive pregnant women and 23/349 (6.59%) HIV-negative pregnant women were HBsAg positive. HBV prevalence in these two groups was not statistically different (p = 0.2). Because of sample exhaustion, 45 out of 51 HBsAg-positive samples were tested for both HBeAg and anti HBe respectively. None of the 45 samples tested for HBeAg was positive.

## Discussions

The results of this study indicate a high prevalence of HBV infection among pregnant women in Cameroon. To the best of our knowledge, this is the first study carried out among pregnant women in an urban setting in Cameroon. Earlier studies had reported an HBsAg prevalence of 5.4% among pregnant women in a rural setting in Cameroon [10]. In a previous study in a general population of Cameroon, about 9.6% of the 272 individuals tested were HBsAg-positive [11]. Another study on city school children reported a high HBsAg prevalence of 19.9% among the 702 school children tested [12]. This high prevalence in children could be due in part to mother-to-child transmission and also could be due to horizontal transmissions between school children [13]. The high prevalence of HBsAg observed in this study among pregnant women could be an indication that pregnant women serve as a very important reservoir to fuel the HBV epidemic in the general population. However, previous studies indicate that in areas of high HBV prevalence (i.e. ≥ 8%) like Cameroon, transmission is said to be predominantly during childhood [14,15].

In our survey, HBsAg positivity rate was similar in HIV-positive (9%) and negative pregnant women (7%). Similar results have been obtained in Côte d'Ivoire [16] a sub-Saharan African country just like Cameroon. This high prevalence among HIV-positive and HIV-negative pregnant women suggests that HBsAg screening should be included as part of prenatal testing in Cameroon. The role of sexual transmission could also be postulated in this population of pregnant women in conformity to what has been previously reported in Tanzania [17].

Hepatitis B 'e' status and viral load are factors associated with the frequency of vertical transmission. Despite the fact that similar and high prevalence of HBsAg was observed in this study, no HBeAg reactive sample was detected in this study population. HBeAg reactive women are known to have a high viral load and to transmit HBV to their children. The rate of maternofetal infection in East Asia, particularly China, was estimated to be about 88% [18], compared with 8% or less observed in the studies conducted in sub-Saharan Africa [19-22]. This difference was largely attributed to the natural history of HBV infection in South-East Asia where infected individuals carry HBeAg and high viral load in age groups that include most women of gestational age [23,24]. Conversely, in sub-Saharan Africa, seroconversion to anti-HBe occurs before age 15 or 16, with the consequence that most women of gestational age carry anti-HBe [25]. Since perinatal transmission of HBV is mostly effective when the mother is HBeAg-positive, this form of transmission could play a negligible role in HBV transmission in Cameroon. Despite the fact that this study did not test for mother-to-child transmission of HBV we could conjecture that horizontal transmissions could be the most common mechanism of HBV infection in Cameroon, as reported in some sub-Saharan African countries [20-22]. Before the integration of HBsAg screening into the routine package of biological analyses carried out during pregnancy, we presuppose that our results are in line and could be considered as a justification of the current immunization program. The present Cameroon vaccination program against HBV consists of vaccination of all newborn at 6 weeks, efforts should therefore be directed at making available these vaccines to all children in Cameroon from 6 weeks old.

We are considering a follow-up of these findings on a larger scale where more pregnant women would be screened with the principal aim of determining the real proportion of HBeAg positive pregnant women and the ensuing mother to child transmission rates of HBV in Cameroon.

## Abbreviations

Anti-HBe: Antibodies to hepatitis B "e" antigen; DNA: Deoxyribonucleic acid; HBeAg: Hepatitis B "e" antigen; HBsAg: Hepatitis B surface antigen; HBV: Hepatitis B virus; HIV: Human immunodeficiency virus; MTCT: Mother-to-child transmission; PHPP: Public health pilot program.

## Competing interests

The authors declare that they have no competing interests.

## Authors' contributions

AKWK designed the study, carried out the immunoassays, drafted the manuscript and participated in the interpretation of data. MCT participated in study design drafted the manuscript and performed the statistical analysis. RN participated in study design, critical revision of manuscript. AKWK, MCT and RN have given final approval of the version to be published. All authors read and approved the final manuscript.

## Authors' information

AKWK holds a PhD in Virology and has been working on mother-to-child transmission of HIV. He is also involved in evaluation of immune responses after vaccination in HIV infected children.

MCT is an MD with an MPH in Epidemiology and Public Health. His works principally involve Mother-to-child transmission of HIV, vaccine responses in children as well as studies on the rabies virus. RN holds a PhD in Virology and has extensively worked on viral hepatitis in both human and non-human primates. He has also worked both on the Avian and human flu.
